# Supplementary material for: Inhibition of AXL and VEGF-A Has Improved Therapeutic Efficacy in Uterine Serous Cancer
Source: Cancers (Basel). 2021 Nov 23;13(23):5877. doi: 10.3390/cancers13235877 (PMC8656641; doi:10.3390/cancers13235877)
Supplement: Supplementary file 1 [file cancers-13-05877-s001.zip › cancers-1427897-supplementary.pdf]

Supplementary Materials

Inhibition of AXL and VEGF-A Has Improved Therapeutic Efficacy in Uterine Serous Cancer

Michael D. Toboni, Elena Lomonosova, Shaina F. Bruce, Jo'an I. Tankou, Mary M. Mullen, Angela Schab, Alyssa Oplt, Hollie Noia, Danny Wilke, Lindsay M. Kuroki, Andrea R. Hagemann, Carolyn K. McCourt, Premal H. Thaker, Matthew A. Powell, Dineo Khabele, David G. Mutch and Katherine C. Fuh

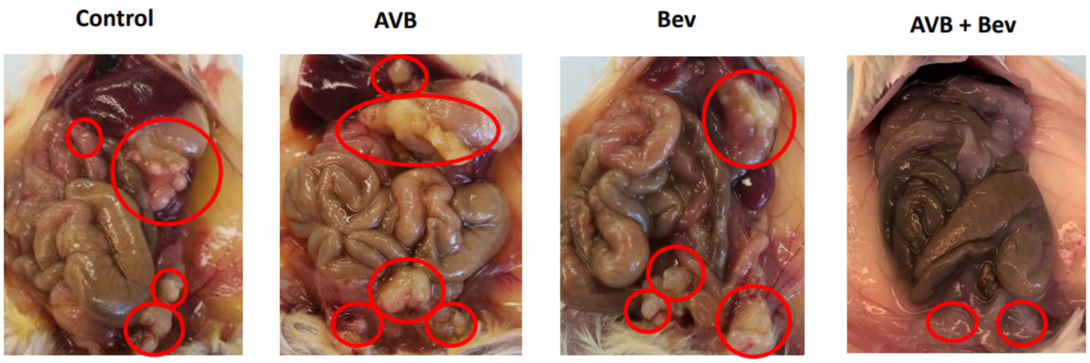

Figure S1. Representative images from the in vivo experiment. Red circles indicate tumor burden visualized intraperitoneally. AVB+Bev mouse has tumor limited to the pelvis with no upper abdominal disease visualized in comparison to the other treatment conditions. .

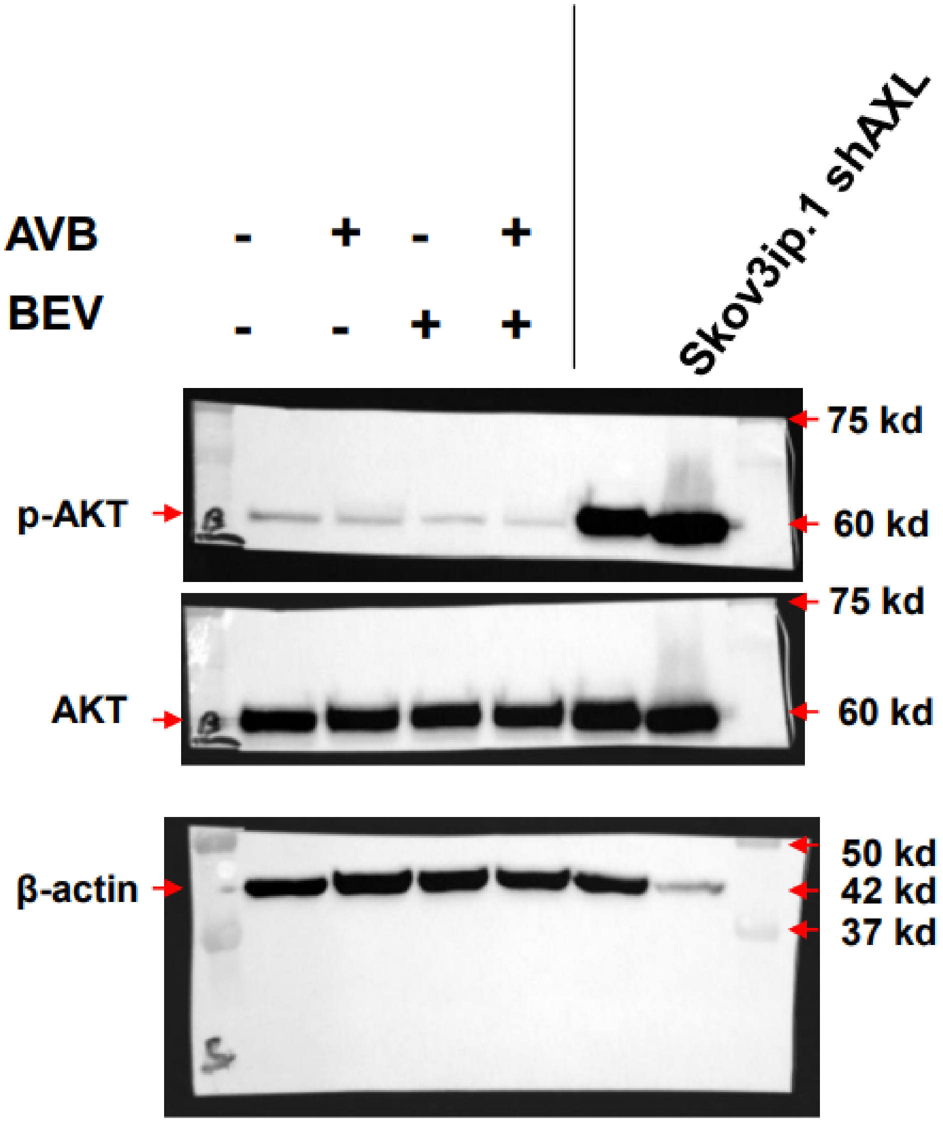

Figure S2. Complete original Western blot.
